# Performance in grade 12 mathematics and science predicts student nurses’ performance in first year science modules at a university in the Western Cape

**DOI:** 10.4102/curationis.v40i1.1776

**Published:** 2017-10-26

**Authors:** Katlego D.T. Mthimunye, Felicity M. Daniels

**Affiliations:** 1 School of Nursing, University of the Western Cape, South Africa

## Abstract

**Background:**

The demand for highly qualified and skilled nurses is increasing in South Africa as well as around the world. Having a background in science can create a significant advantage for students wishing to enrol for an undergraduate nursing qualification because nursing as profession is grounded in scientific evidence.

**Aim:**

The aim of this study was to investigate the predictive validity of grade 12 mathematics and science on the academic performance of first year student nurses in science modules.

**Method:**

A quantitative research method using a cross-sectional predictive design was employed in this study. The participants included first year Bachelor of Nursing students enrolled at a university in the Western Cape, South Africa. Descriptive and inferential statistics were performed to analyse the data by using the IBM Statistical Package for Social Sciences versions 24. Descriptive analysis of all variables was performed as well as the Spearman’s rank correlation test to describe the relationship among the study variables. Standard multiple linear regressions analysis was performed to determine the predictive validity of grade 12 mathematics and science on the academic performance of first year student nurses in science modules.

**Results:**

The results of this study showed that grade 12 physical science is not a significant predictor (*p* > 0.062) of performance in first year science modules. The multiple linear regression revealed that grade 12 mathematics and life science grades explained 37.1% to 38.1% (*R*^2^ = 0.381 and adj *R*^2^ = 0.371) of the variation in the first year science grade distributions.

**Conclusion:**

Based on the results of the study it is evident that performance in grade 12 mathematics (*β* = 2.997) and life science (*β* = 3.175) subjects is a significant predictor (*p* < 0.001) of the performance in first year science modules for student nurses at the university identified for this study.

## Introduction

Higher education institutions in South Africa are challenged on how to produce quality students in a constantly increasing globalised and competitive environment (Harvey & Kamvounias 2008). However, not all students admitted to an undergraduate nursing programme will meet the academic expectations and minimum requirements stipulated to complete the programme.

The literature contains studies on factors that influence academic performance and the throughput rate of undergraduate student nurses over the past few decades (Abele, Penprase & Ternes 2013; Everett et al. 2013; Glew et al. 2015; Jeffreys 1998, 2001, 2007, 2012; Kowitlawakul, Brenkus & Dugan 2013). However, in South Africa further evidence could help to identify whether performance in grade 12 mathematics and science subjects is correlated with student nurses’ academic performance.

According to the South African Nursing Council (SANC), Regulation 425 of the *Nursing Act 33* of 2005, as amended, general nursing science (GNS) should be a compulsory module in the nursing curriculum and should form the foundation of nursing science which requires an understanding of anatomy, physiology, pathophysiology, physics, chemistry and pharmacology. GNS should also be integrated with social and biological science. This statement implies that mathematics and science subjects are highly recommended for understanding the basis of nursing and for success in GNS (Wolkowitz & Kelley 2010). However, in South Africa little evidence has been published on whether students who are admitted to Bachelor of Nursing programmes require a mathematics and science background based in the likelihood of improving success in their nursing studies.

The entry requirements for the Bachelor of Nursing programme at the university participating in this study include: level 4 (50% – 59%) in English (home or first additional language) and level 3 (40% – 49%) in another language (home or first additional language), level 4 (50% – 59%) in life science and level 3 (40% – 49%) in mathematics or level 4 (50% – 59%) in mathematical literacy. This study investigated the predictive validity of grade 12 mathematics and science subjects on the academic performance of first year student nurses in science modules at a university in the Western Cape, South Africa.

### Significance of the study

The scientific evidence obtained in this study may provide nursing education institutions with significant information for consideration in the refinement of their admission or selection criteria for the Bachelor of Nursing programme. The results may also assist nurse educators to identify and support first year student nurses who are at risk of unsatisfactory academic performance.

### Literature and theoretical framework

Many higher education institutions depend on the admission criteria to recruit high-quality prospective students. Admission officers at these higher education institutions are tasked with the challenging responsibility of distinguishing who would potentially be successful in the programme they wish to register for. The same applies to nursing programmes worldwide (Hopkins 2008). Traditionally it was viewed as highly probable that students who performed well during grade 12 would perform better in undergraduate programmes than those students who did not do well (Hopkins 2008). Hence, performance in grade 12 is still viewed as an important indicator for selecting students who are potentially ready for undergraduate studies.

Some studies found address the impact of cognitive variables such as high school grades as well as pre-admission aptitude tests on the academic performance and success of student nurses (Aiken, Cervero & Bailey 2001; Campbell & Dickson 1996; Jeffreys 2007). Several studies have been conducted around the world which revealed that a science background has a significant impact on the academic performance as well as success in nursing programmes (Lewis & Lewis 2000; Wolkowitz & Kelley 2010; Wong & Wong 1999). Frost (2004), for example, reported that mathematics skills are essential for academic success in the healthcare professions. This can be understood in terms of the need for doctors and nurses to calculate, often using mathematical equations to calculate drug dosages and monitor changes in the patients’ health. In South Africa, however, research that the researcher is aware of has not been decisive regarding the influence of grade 12 mathematics and science on the performance of student nurses.

The theoretical framework used in this study is based on Jeffreys’s model of Nursing Undergraduate Retention and Success (NURS) and sheds light on the relationship between the identified variables (Jeffreys 2015). According to Jeffreys (2015), the performance and ultimately the success and retention of student nurses are an intricate and multidimensional phenomenon. Jeffreys maintained that students’ academic success and retention outcomes are grounded on the interaction of student profile characteristics, student affective factors, academic factors, environmental factors, professional integration factors, academic outcomes, psychological outcomes and outside surrounding factors (Jeffreys 2015). It was, however, not within the scope of the current study to test the entire NURS model. For this study the NURS model was used to investigate the relationship between grade 12 mathematics and science and academic performance of first year Bachelor of Nursing students at a university in the Western Cape.

## Methods

### Design

A quantitative research method with a cross-sectional predictive design was used in this study to determine the predictive validity of grade 12 mathematics and science on the academic performance of first year student nurses in science modules at the identified university.

### Research question

Can the academic performance of student nurses in first year science modules be predicted by their performance in grade 12 mathematics and science subjects?

### Population and sampling

The study population (*N* = 543) comprised two cohorts of students. The first cohort completed their first year during 2012 (*N*_1_= 277; 51%) and the second cohort completed their first year during 2013 (*N*_2_ = 266; 49%). This population provided the latest data of student nurses after the implementation of the National Senior Certificate (NSC) as certified by Umalusi^1^ (UMALUSI 2009). All-inclusive sampling was used after applying the inclusion and exclusion criteria. The total sample (*n* = 226) for the study therefore comprised the sum of the sample obtained from the first cohort (*n*_1_ = 101) and from the second cohort (*n*_2_ = 125).

### Instrument and data collection procedure

Data were collected from the student data base, referred to as Student Administration System Integrated (SASI), at the identified university during February 2015. The relevant data were extracted, by personnel in the student administration office, from the final grade 12 data submitted by students as reflected on their Umalusi matriculation certificates and captured on SASI. The researcher, with the help of a research assistant, extracted the following data, using a checklist, to enable the researcher to answer the research question.

### Data analysis

Descriptive and inferential statistical analyses were performed by using the IBM Statistical Package for Social Science (IBM SPSS-24). Spearman’s rho correlational analyses were performed to determine the relationship between the study variables (Creswell 2014). The non-parametric Spearman’s rho correlational analyses were appropriate for this study mainly because data for independent variables were categorical and not normally distributed (Field 2013).

This type of correlational statistics is useful because it can be used for both categorical and continuous data (Field 2013). Standard multiple linear regression analysis was performed to answer the research question and to determine the predictive validity of grade 12 science subjects in predicting the performance of Bachelor of Nursing students in their first year of study.

### Rigour

A statistician and the study supervisor were consulted to confirm the appropriateness and the accuracy of the instrument. A pre-test of the instrument was conducted to test the feasibility of the checklist used to collect data from SASI to establish the inter-rater reliability (De Vos et al. 2011).

### Ethical consideration

Approval to conduct the study was granted by the research ethics committee of the university used for the study (Registration No. 14/9/38). The Registrar and the Director of the School of Nursing granted permission for the study to be conducted. The researcher maintained the principle of anonymity and confidentiality throughout the study (Grove, Burns & Gray 2012).

## Results

### Independent predictor variables

#### Grade 12 life science grade

The ordinal scale for grade 12 life science grade ranged from level 3 to level 7 with a mode of 5 and a median of 5. The mean was calculated and found to be 4.9 with a standard deviation of 0.9. A grade 12 life science grade was missing for one student.

#### Grade 12 physical science grade

The ordinal scale for grade 12 physical science grade ranged from level 1 to level 7 with a mode of 4 and median of 4. The mean was found to be 3.6 with a standard deviation of 1.2. A grade 12 physical science grade was missing for 70 students. Students with no grade in this subject are likely, by choice, not to have registered for this subject in grade 12. Although this subject is recommended and not a required subject for admission to the Bachelor of Nursing programme, it was deemed necessary to test based on the probability of it having significant influence on the performance of student nurses in science modules.

#### Grade 12 mathematics grade

The ordinal scale for grade 12 physical science grade ranged from level 3 to level 7 with a mode of 3 and median of 4. The mean of 3.8 was calculated with a standard deviation of 0.9.

### Outcome variables

#### First year science modules

The first year science module grades were obtained by calculating the average grade between the following first year science modules: human biology (HUB 118 and HUB 128), physics for community and health science students (PHY 118) and chemistry (CHM 128). The first year grade for science modules ranged from 43.3% to 90% with a mode of 57.5% and a median of 64.5%. The mean for this outcome variable was 64.7% with a standard deviation of 9.2.

### The results of correlational analysis

The Kolmogorov–Smirnova (*p* < 0.001) and Shapiro–Wilk (*p* < 0.001) tests performed indicate that the independent variable for this study is not normally distributed, and therefore the non-pragmatic test is appropriate (Field 2013). For the outcome variables the Kolmogorov–Smirnov^a^ (*p* < 0.05) and Shapiro–Wilk (*p* > 0.05) tests indicate that the values are normally distributed, and therefore the pragmatic test is appropriate (Field 2013). Table 1 summarises the normality test of the study variables.

**TABLE 1:** Tests of normality.

Variables	Kolmogorov–Smirnov^a^			Shapiro–Wilk		
	Statistic	df	Sig.	Statistic	df	Sig.
Life science grade	0.232	135	0.000	0.853	135	0.000
Physical science grade	0.228	135	0.000	0.920	135	0.000
Mathematics grade	0.262	135	0.000	0.746	135	0.000
First year science grade	0.062	135	0.200*	0.985	135	0.152

*Source*: Authors’ own work

Sig., Significance; df, degrees of freedom.

* This is a lower bound of the true significance.

a Lilliefors significance correction.

The results of the Spearman correlation (*r*_*s*_) analysis (Table 2) indicate a significant positive correlation between the following variables: physical science and first year science modules (0.522, *p* < 0.01); life science and first year science modules (0.478, *p* < 0.01); and mathematics and first year science modules (0.464, *p* < 0.01). Spearman correlation coefficients revealed that there are no significantly large (+ 0.6) relationships between any of the independent predictor variables. Significant correlations among the predictor variables were found between physical science and life science (0.523, *p* < 0.01); physical science and mathematics (0.436, *p* < 0.01); and life science and mathematics (0.350, *p* < 0.01).

**TABLE 2:** Spearman’s rank order correlations between study variables.

Spearman’s rank order correlation coefficient	Life science	Physical science	Mathematics	First year science
Life science				
Correlation coefficient	1.000	0.523**	0.350**	0.478**
Sig. (two-tailed)	-	0.000	0.000	0.000
*N*	225.000	156.000	153.000	225.000
Physical science				
Correlation coefficient	0.523**	1.000	0.436**	0.522**
Sig. (two-tailed)	0.000	-	0.000	0.000
*N*	156.000	156.000	135.000	156.000
Mathematics				
Correlation coefficient	0.350**	0.436**	1.000	0.464**
Sig. (two-tailed)	0.000	0.000	-	0.000
*N*	153.000	135.000	154.000	154.000
First year science				
Correlation coefficient	0.478**	0.522**	0.464**	1.000
Sig. (two-tailed)	0.000	0.000	0.000	-
*N*	225.000	156.000	154.000	226.000

*Source*: Authors’ own work

** Correlation is significant at the 0.01 level (two-tailed).

### Collinearity test

The collinearity test results (Table 3) indicated the tolerance well above 0.2 and the variance inflation factors (VIF) are below 2.5 for all of the variations of the model, which implies that there were no problems related to multicollinearity within the data.

**TABLE 3:** Collinearity test (coefficients^a^).

Model (Constant)	Collinearity statistics	
	Tolerance	VIF
Life science grade	0.686	1.457
Physical science grade	0.610	1.639
Mathematics grade	0.747	1.339

Source: Authors’ own work

a Dependent variable: First year science grade; VIF, variance inflation factors.

### Results of multiple regression analyses of first year

Tables 4 and 5 present the module summaries indicating the predictive validity of predictor variables (grade 12 mathematics and science subjects) with grade for first year science modules as the dependent variable. The method that was used to assess the variations in the outcome variable was generalised *R* square (*R*^2^) statistics. In multiple regressions, *R*^2^ measures the amount of variance in the outcome variable.

**TABLE 4:** Multiple regression model summary predicting grade for first year science modules.

Variable	Coefficients	Standard error	*t* stat	*p*
Intercept	42.649	3.150	13.538	0.000
Life science grade	2.583	0.700	3.692	0.000
Physical science grade	1.105	0.587	1.885	0.062
Mathematics grade	2.566	0.631	4.065	0.000

*Source*: Authors’ own work

Overall model: *R*^2^ = 0.397, *F*(df) = 28.769 (3), *p* < 0.000, adjusted *R*^2^ = 0.383.

**TABLE 5:** Multiple regression model summary predicting grade for first year science modules.

Variable	Coefficients	Standard error	*t* stat	*p*
Intercept	42.196	3.171	13.305	0.000
Life science grade	3.175	0.631	5.029	0.000
Mathematics grade	2.997	0.594	5.045	0.000

*Source*: Authors’ own work

Overall model: *R*^2^ = 0.381, *F*(df) = 40.593 (2), *p* < 0.000, adjusted *R*^2^ = 0.371.

Model 1 (Table 4) performs a standard multiple linear regression analysis between the student nurses’ academic performance in first year science modules as the outcome variable and all three grade 12 subjects (life science, physical science and mathematics) selected for this study as predictor variables. Although physical science was found to be correlated with the grade for first year science modules (0.478, *p* < 0.01), the results of model 1 revealed that physical science was not a significant predictor of student nurses’ academic performance in first year science modules (*p* > 0.05).

Model 2 (Table 5) performs a standard multiple linear regression analysis between the student nurses’ academic performance in first year science modules as the outcome variable and only two of the selected grade 12 subjects (life science and mathematics) as predictor variables. The results of this model revealed that both life science and mathematics were significant predictors (*p* < 0.001) of academic performance. The generalised *R*^2^ = 0.381 (*p* < 0.001) and the adjusted *R*^2^ of 0.371 (*p* < 0.001) suggest that model 2 explains 37.1% to 38.1% of the variation in the first year science grade distributions. According to beta weight results (beta coefficients results) both life science (*β* = 3.175, *p* < 0.001) and mathematics (*β* = 2.997, *p* < 0.001) contributed significantly towards the model. The coefficient (*β* = 3.175, *p* < 0.001) for life science indicates that for every additional level in life science grades, an increase by an average of 3.175% in first year science module grades can be expected. Likewise, the coefficient (*β* = 2.997, *p* < 0.001) for mathematics indicates that every additional level in grade 12 mathematics will result in a nearly 3% increase in first year science module grades.

## Discussion

The aim of this study was to investigate the predictive validity of grade 12 mathematics and science on the academic performance of first year student nurses in science modules at the participating university in the Western Cape, South Africa. Many nursing schools indicate mathematics and science subjects as admission or selection criteria for undergraduate nursing programmes. At the participating school of nursing, life science and mathematics are compulsory subjects while physical science is a recommended subject for admission. In the first year students enrol for four science modules (HUB 118, HUB 128, PHY 118 and CHM 128), which form the basis for understanding nursing science. These modules are offered on a full-time basis. The students with a mathematics and science background may be in an advantaged position compared to their counterparts who do not have these grade 12 subjects.

### Life science

The results of this study shows that grade 12 life science grades have a significant (*p* < 0.01) relationship with grades in first year science modules (*r*_*s*_ = 0.478), physical science (*r*_*s*_ = 0.523) and mathematics (*r*_*s*_ = 0.350). Grade 12 life science is also a significant predictor (*β* = 3.175, *p* < 0.001) of academic performance in first year science modules. Similar to a study conducted by Symes, Tart and Travis (2005), life science was found to be significantly correlated with academic performance as well as success of student nurses. Likewise, in the study conducted by Alden (2008), life science grade was found to be a significant predictor of early academic success among Bachelor of Nursing students.

### Mathematics

The results of this study show that grade 12 mathematics has a significant (*p* < 0.01) relationship with the grade in first year science modules (*r*_*s*_ = 0.464), physical science (*r*_*s*_ = 0.436) and life science (*r*_*s*_ = 0.350). Grade 12 mathematics is also a significant predictor (*β* = 2.997, *p* < 0.001) of academic performance in first year science modules. Frost (2004) also reported that mathematics is critical for success in the healthcare professions, including nursing programmes. Previous studies such as those by Brennan, Best, and Small (1996) and Hayes (2005) found mathematics to be significantly correlated (*p* < 0.01) to grades in nursing modules, pharmacology and science modules such as physics.

### Physical science

There are significant correlations (*p* < 0.01) between physical science and first year science modules (*r*_*s*_ = 0.522), life science (*r*_*s*_ = 0.523) and mathematics (*r*_*s*_ = 0.436). Hayes (2005) found that student nurses with a background of physical science outperformed those without a background of physical science. In contrast, the results from this study suggest that physical science subject is not a significant predictor (*p* > 0.062) of performance in first year science modules. Although physical science was not statistically significant (*p* > 0.05) the influence that physical science has on first year science grades is still noted in model 1 (*R*^2^ = 0.397) as compared to model 2 (*R*^2^ = 0.381).

The fact that physical science grade was missing for 70 students as it is not compulsory for admission to the Bachelor of Nursing programme can explain this result. Further research to examine the predictive validity of physical science on the academic performance of student nurses is highly recommended.

## Limitations

The study was conducted with a limited sample from one university. The sample comprised only 226 students, which may limit the generalisability of the results to nursing schools at other universities. Furthermore, physical science grade was missing for 70 students which may have had a negative influence on the results.

## Recommendations

The researcher recommends further research where the sample is more diverse and includes more nursing schools to increase the generalisability of the results.

Students with less satisfactory grades in grade 12 science subjects may be considered for the extended nursing programme, or if admitted to the mainstream nursing programme, they should be monitored closely and be provided with the needed academic support.

## Conclusion

Students’ performance in grade 12 mathematics and life science subjects was found to be a significant predictor of first year student nurses’ academic performance in science modules. The NURS model provided a framework for understanding the complex process of nursing students’ retention and success. The study tested the parts of the NURS model as defined by Jeffreys (2015). Grade 12 mathematics and science variables in this study were representative of the student profile characteristics (prior educational experiences) in the NURS model. Therefore, the findings of the study support the importance of prior educational experience in forecasting the likelihood that the student will successfully complete the programme.

## Footnotes

1 malusi, the Council for Quality Assurance in General and Further Education and Training, South Africa.
